# Extraction of Lipids from Liquid Biological Samples for High-Throughput Lipidomics

**DOI:** 10.3390/molecules25143192

**Published:** 2020-07-13

**Authors:** Samuel Furse, Adam J. Watkins, Albert Koulman

**Affiliations:** 1 Core Metabolomics and Lipidomics Laboratory, Wellcome Trust-MRC Institute of Metabolic Science, University of Cambridge, Box 289, Cambridge Biomedical Campus, Hills Road, Cambridge CB2 0QQ, UK; 2 Division of Child Health, Obstetrics and Gynaecology, Faculty of Medicine, University of Nottingham, Nottingham NG7 2UH, UK; adam.watkins@nottingham.ac.uk

**Keywords:** lipidomics, lipid extraction, lipid metabolism

## Abstract

Extraction of the lipid fraction is a key part of acquiring lipidomics data. High-throughput lipidomics, the extraction of samples in 96w plates that are then run on 96 or 384w plates, has particular requirements that mean special development work is needed to fully optimise an extraction method. Several methods have been published as suitable for it. Here, we test those methods using four liquid matrices: milk, human serum, homogenised mouse liver and homogenised mouse heart. In order to determine the difference in performance of the methods as objectively as possible, we used the number of lipid variables identified, the total signal strength and the coefficient of variance to quantify the performance of the methods. This showed that extraction methods with an aqueous component were generally better than those without for these matrices. However, methods without an aqueous fraction in the extraction were efficient for milk samples. Furthermore, a mixture containing a chlorinated solvent (dichloromethane) appears to be better than an ethereal solvent (*tert-*butyl methyl ether) for extracting lipids. This study suggests that a 3:1:0.005 mixture of dichloromethane, methanol and triethylammonium chloride, with an aqueous wash, is the most efficient of the currently reported methods for high-throughput lipid extraction and analysis. Further work is required to develop non-aqueous extraction methods that are both convenient and applicable to a broad range of sample types.

## 1. Introduction

Lipidomics is of increasing interest in metabolic and other biological studies. Trials comprising sizeable numbers of samples are now being attempted in order to provide sufficient statistical power to answer questions about human and animal metabolism, development and dysregulation of metabolism, and development [1,2,3,4,5,6], as well as in particular sample types [7]. This has encouraged research efforts to overcome the practical concerns that pertain to determining the lipid composition of biological samples (lipidomics). This comes against a background of investigations of a variety of sample formats and scales, including mammalian plasma [8,9,10,11,12,13], fibrous tissues [14,15,16], dried blood spots [17,18,19] and even milk [20,21]. General methods for isolating lipids for low- and medium-throughput studies have been reviewed [22,23].

However, due to its relatively recent arrival and particular needs, high-throughput lipidomics and methods associated with it are less widely researched. Despite that, considerable progress has been made in recent years in laboratory infrastructure and methods for the high-throughput lipidomics required for large human trials. Spectroscopic innovations such as Direct Infusion Mass Spectrometry (DI-MS) [21,24] and dual spectroscopy [14] have been published. Lipid extraction methods for the particular challenges of high-throughput lipidomics have also been developed, such as those by Maytash et al. [8] and the Meikle group [9,10], and from our own laboratory [14,25].

However, questions and challenges about high-throughput lipidomics remain. Some of these are general questions about sample handling and lipid extraction that have existed for some time [22]. These include problems with the suitability of solvents for the lipid species of interest, chemical degradation associated with particular solvents and the activity of endogenous enzymes *ex vivo.* However, some questions are unique to high-throughput lipidomics. For example, there is a clear need to find a compromise between the practicalities of large numbers of simultaneous extractions of lipid material from small biological samples and the desire for thorough molecular profiling. Some of these problems have been solved by using glass-coated deep-well plates accompanied by 96 channel pipettes mounted on a movable platform for liquid transfer and mixing. However, the need to strike a compromise between a large number of samples, analytical soundness and acquiring quantitative data relevant to hypotheses has led to the development of at least four methods of extracting lipids for high-throughput lipidomics using mass spectrometry. However, it is not clear how they perform when compared to one another. This is partly due to the lack of an objective means for measuring extraction efficiency, but also because the reported methods are distinct from one another. For example, there are two categories of extraction, those that comprise an aqueous wash as part of the process and those that do not. The absence of an aqueous wash is attractive in practical terms, as it simplifies sample preparation for chromatography or infusion. However, it is not clear whether this approach can be applied to more proteinaceous or more viscous sample matrices. This led us to the hypothesis that a solvent-based extraction method that comprises an aqueous wash and the facility to dissolve lipid classes with a range of polarities works best for high-throughput lipidomics.

In order to test this hypothesis, we investigated for four lipid extraction methods reported for high-throughput lipidomics on four distinct matrices. The four lipid extractions were ‘TBME’ (*tert-*butylmethyl ether) [8], ‘BuMe’ (butanol/methanol, 1:1) [9,10], ‘DMT’ (dichloromethane/methanol/triethylammonium chloride, 3:1:0.005) [14,21,26] and ‘XMI’ (xylene/methanol/isopropanol, 1:2:4) [25]. These methods are in common use for lipidomics and have shown reliability with at least one sample type. The TBME method [8] was amongst the first to be reported for high-throughput lipidomics, and has received wide attention and considerable use in lipidomics. It has been used particularly frequently in plasma and serum sample sets, and has been found to be consistent. DMT has been reported more recently, has been used across a number of sample types and has been used where several sample types are required in a given study [14,21]. BuMe was developed solely for human plasma samples, and has not yet been reviewed for other sample types [9,10]. XMI is the newest method, developed by us for isolating the lipid fraction from dried milk spots, and it too is untested on other matrices [25]. Notably, DMT and TBME are both aqueous methods, they both comprise a wash with water as part of sample preparation. BuMe and XMI methods involve dispersing the biological sample into the solvent mixture before infusion into the ion source.

The four matrices used in this study were bovine milk (milk), human serum (serum), murine liver homogenate (liver) and murine heart homogenate (heart). These were chosen to represent the breadth of biological samples commonly requested in lipidomics, with heart representing a proteinaceous sample and milk representing sample types with a particularly high proportion of triglycerides (phospholipid:triglyceride = 1:49). Sets of subtypes or pools of these sample matrices were used to assess the precision of the methods. The data for this study were collected in one analytical run using Direct-Infusion Mass Spectrometry (DI-MS). This is a typical high-throughput method (30 samples/h) with a sample queue that minimizes or avoids batch effects altogether and thus tests the methods in a high-throughput manner.

It is important to test the hypothesis that the solvent-based extraction method that comprises an aqueous wash and the facility to dissolve lipid classes with a range of polarities works best for high-throughput lipidomics, as understanding the limits of lipid extraction methods is key to choosing the appropriate method for answering scientific questions about lipid metabolism. Questions requiring a focus on one or two particular classes or particular sample matrices may be answered by methods that are particularly amenable to the chemical and physical properties of that class/sample type. It may also be helpful to know which lipid classes are extracted less efficiently using a given isolation method. This study represents an advance because previous attempts at comparing extraction methods have focused on low-resolution profiling and the undried lipid extracts, meaning that data in this area is weak [22]. The present study uses a novel method to quantify the quality of lipid extraction, using both the number of lipid isoforms (variables) identified and the total signal strength of lipid variables the extracts. Isoforms are defined by the configuration of their FA or acylated sphingosinyl portion, i.e., phosphatidylcholine with two palmitate residues will be referred to as the isoform PC(32:0), while that with two arachidonate residues will be referred to as PC(40:8). The combination of signal strength and number of variables provides an objective measure for ranking the efficiency of lipid extraction that has not previously been used in assessing the quality or efficiency of this process.

## 2. Results

### 2.1. Extraction Efficiency

**Figure 1:**
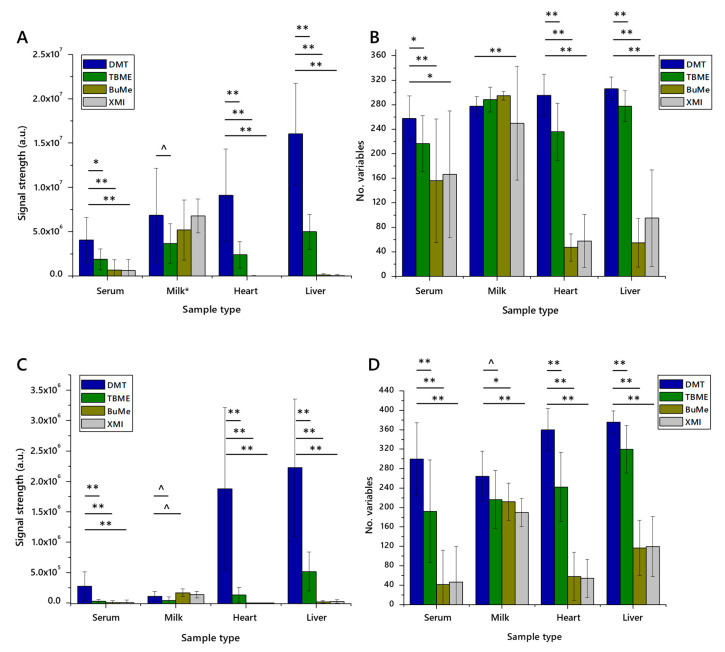
The signal strength and number of lipid variables detected in lipid extracts from four different matrices using four different extraction methods for high-throughput lipidomics (*n* = 20 measurements of each). Panel (**A**), total signal strength in positive ionisation mode; (**B**), number of variables identified in positive mode; (**C**), total signal strength in negative ionisation mode; (**D**), number of variables identified in negative mode. Error bars show standard deviation. BuMe, butanol/methanol 1:1; DMT, dichloromethane/methanol/triethylammonium chloride 3:1:0.005; TBME, *tert*-butylmethylether; XMI, xylene, methanol, isopropanol 1:2:4. Samples: serum, human blood serum; milk; unhomogenised Jersey cows’ milk; heart, murine heart homogenate; liver, mouse liver homogenate. ^ *p* < 0.05; * *p* < 0.01; ** *p* < 0.001.

Twenty measurements, each of the four methods (DMT [14,21,26], TBME [8], BuMe [9,10] and XMI [25]) across the four matrices (JerseyMilk, serum, liver and heart), were taken. Strikingly, the Jersey Milk matrix was extracted efficiently across all methods tested, and in both ionisation modes. The number of variables was highest in extracts using BuMe with the highest signal strength using the DMT method (Figure 1).

Aqueous methods (DMT and TBME) performed best across all matrices, with particular superiority in proteinaceous tissue homogenates (liver, heart), as shown in Figure 1. Sample preparation using non-aqueous methods, XMI and BuMe, was quick and straightforward. However, the mass spectrometry infusions that of XMI and BuMe samples were characterised by poor injection performance. This is ascribed to the high protein abundance in those samples. It should be stressed, however, that XMI and BuMe were developed for dried milk spots and plasma, respectively, and not high-protein samples. This may also explain the weaker performance of these methods on serum, which is more viscous than the plasma that the BuMe method was developed for.

The TBME and DMT methods performed better than the non-aqueous methods, presumably assisted by the aqueous fraction’s ability to dissolve and separate proteinaceous and perhaps carbohydrate material from the organically-soluble fraction. The isolates prepared using the DMT method comprised more variables and gave rise to a higher total signal strength than those prepared using TBME.

**Figure 2:**
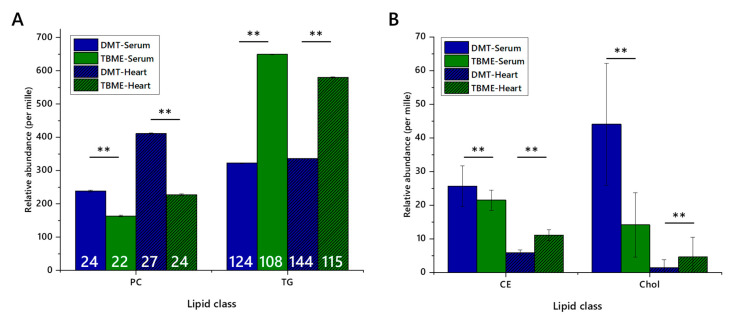
Relative abundance of lipid classes and the numbers of lipid isoforms (variables) in each class, extracted from either murine heart or human serum using either TBME or DMT. Blue columns show DMT extracts, and green columns show TBME extracts. Hatched columns represent serum samples, whereas open columns represent heart samples. Number of variables are shown in white figures. Panel (**A**), abundance of phosphatidylcholines and triglycerides with number of variables marked; (**B**), abundance of cholesteryl esters and cholesterol. Five isoforms of cholesteryl ester were identified. Error bars show standard error. CE, cholesteryl ester; Chol, cholesterol; PC, phosphatidylcholine; TG, triglyceride. ** *p* < 0.001.

Whether or not class abundance differed between extraction methods was then tested by assessing the relative abundance of representative lipid classes. A major lipophilic component (triglycerides) and an abundant zwitterionic phospholipid (phosphatidylcholine), as well as a lower abundance lipophilic class, sterols (cholesteryl esters and cholesterol itself) in serum and heart preparations, Figure 2, were used. These classes represent the bulk of the signal strength recorded, and are of interest in metabolic studies and thus are important in deciding which method(s) are most appropriate or efficient for a given study. The relative abundance scores of phosphatidylcholine (PC), triglycerides (TGs), cholesteryl esters (CEs) and cholesterol indicated that the relative abundance of PC was higher and TG lower in DMT extractions. Further, the number of variables was higher for DMT extractions, as was the total signal strength. Importantly, all four of these classes dissolved easily in dichloromethane and dichloromethane-methanol mixtures, with ionic species dissolving less well in ethereal solvents.

The suggestion that isolates of lipids from a range of pipettable biological samples, prepared using DMT, were more concentrated and contained more species of interest than those of other methods led us to test the precision of the method.

### 2.2. Coefficient of Variance

In order to test the precision of the DMT method, four subtypes of each matrix were employed. Commercially-available milks from four different sources (Jersey and ordinary bovine milk, caprine milk and soya drink), and two pools of homogenates prepared from tissues from mice of two different feeding phenotypes, were used.

**Figure 3:**
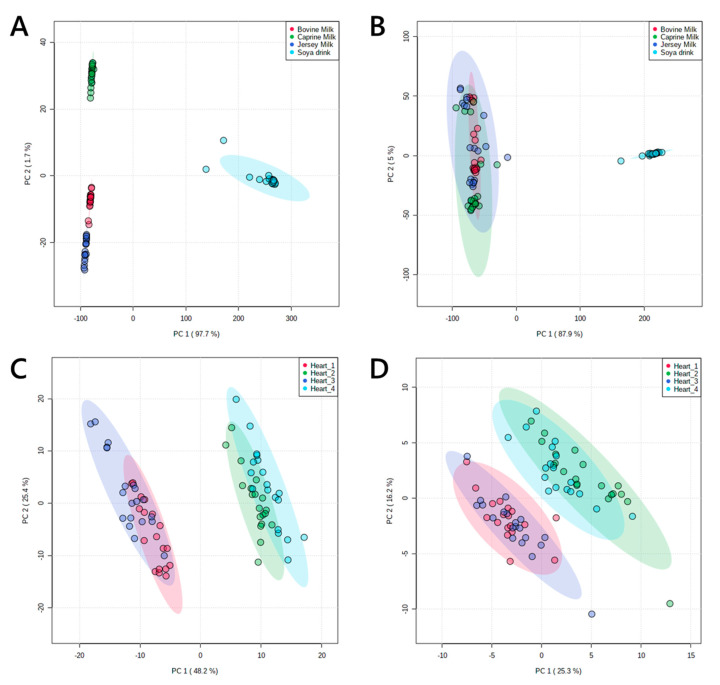
Principal component analyses of lipid signals from organically-soluble extracts of pooled murine hearts and four animal milks. Panel (**A**), lipid signals from commercial animal milks in positive ionisation mode; (**B**), lipid signals from commercial animal milks in negative ionisation mode; (**C**), lipid signals from murine hearts in positive ionisation mode; (**D**), lipid signals from murine hearts in negative ionisation mode. Murine hearts were drawn from two pools of individuals each from two phenotypes. Coefficient of variance (CV) values are shown in Table 1 and in Supplementary information.

Multivariate analysis (principal component analysis, PCA in Figure 3) showed that the profiles of these groups divided up as expected, with two broadly similar pairs of profiles of lipids from murine hearts and four distinct groups for milk extracts. The latter was shaped by the difference between soya drink and the three animal milks. However, the difference between them was clear from their profile in positive ionisation mode. This was as expected; around 98% of the lipophilic material extracted from milk is the tri- and diglycerides, which ionise very well in positive mode and only poorly in negative mode.

**Figure 4:**
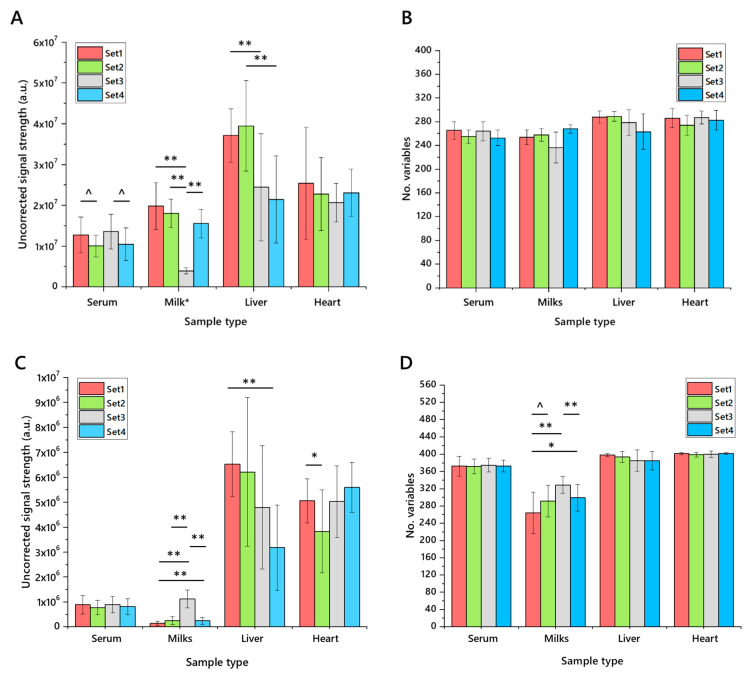
The uncorrected signal strength and number of variables detected in lipid extracts from four different matrices using four different extraction methods for high-throughput lipidomics (*n* = 20 measurements of each). Panel (**A**), total signal strength in positive ionisation mode; (**B**), number of variables identified in positive mode; (**C**), total signal strength in negative ionisation mode; (**D**), number of variables identified in negative mode. Error bars show standard deviation. Extractions were performed using DMT, dichloromethane/methanol/triethylammonium chloride 3:1:0.005 with an aqueous wash. Samples: Serum, human blood serum; Milk (set1, unhomogenised Jersey milk; set2, whole caprine milk; set3, soya drink; set4, whole, homogenised bovine milk); heart, murine heart homogenate; liver, mouse liver homogenate. Murine heart and liver samples were drawn from two pools of two feeding phenotypes. Milk sample values were scaled (reduced by an order of magnitude) to fit. ^ *p* < 0.05; * *p* < 0.01; ** *p* < 0.001.

The same samples were used to characterise the precision of the extraction, both through the number of variables identified, total uncorrected signal strength and the coefficient of variance. The number of variables was relatively consistent across each matrix, as was the total signal strength, with expected variation in milk samples (Figure 4). Some trivial differences between phenotypes were observed. Calculations of the coefficient of variance (CV) showed that around two thirds of variables had a CV of less than 50%, with typically 30–40% of variables having a CV of less than 20%. One notable exception to this is milk samples (especially in negative ionisation mode), which perform less well. This is ascribed to the variation in composition between bovine, caprine and soya sources. In general, across the four matrices, the measurements in positive and negative ionisation modes are consistent, suggesting that the CV for both glyceride isoforms (principally TGs, ionising in positive mode) and anionic/zwitterionic phospholipids is similar.

## 3. Discussion

In this study, it was found that the performance of lipid extraction methods differed considerably between both the format of the extraction (solvent type, use of aqueous wash) and sample type (high/low protein). All methods tested performed well on milk samples, with human serum and tissue homogenates (mouse tissues, heart and liver) being more challenging. Investigation of both total signal and the number of variables observed showed that DMT is a more effective solvent system for isolating the lipid fraction than TBME.

The increased number of variables and apparent mass of material isolated using the DMT method is encouraging for high-throughput lipidomics studies, as it offers greater insight in the molecular composition of the biological system it represents. This also allows researchers to make better use of equipment. However, it does raise questions about how such data should be handled. A typical and very useful approach is to assess lipidomes through a normalised or semi-quantitative abundance, i.e., relative abundance based on signal strength. It is arguable that this is more difficult where more signals are found, as the abundance of any one thus falls. This means that the abundance of less prevalent species may be more difficult to compare between phenotypes. However, such a problem has been common with low-abundance species for some time. This problem may be addressed by following up DI-MS profiling of the lipidome with liquid chromatography-mass spectrometry on a select group of samples, shedding light not only on the comparative abundance of low abundance species between samples but also for isoform analysis of such species [14].

The difference in performance of the DMT and TBME methods naturally raises the question of why this should be. It is well known that chlorinated solvents and others such as ethyl acetate are broad-spectrum solvents, often able to dissolve ionic organic compounds easily. Ethereal solvents are typically better at dissolving more lipophilic compounds, as they are less able to support salts. This suggested to us that a solvent mixture such as DMT may be a good all-round solvent system, where TBME may be better for more lipophilic compounds. The choice of solvent therefore depends upon the question being asked. Certain questions, for example, centring on the TG markers of *de novo* lipogenesis, may be answered by using either DMT or TBME methods, as the species of interest are abundant and expected to dissolve in either solvent. However, where hypotheses are based around changes to several members of a class or several isoforms across classes, a broader-spectrum system may be preferable.

This study was predicated on the notion that lipid extraction efficiency can be measured quantitatively. In a previous report, we collected 16–20 measurements of each sample type or method of interest and used a combination of the number of lipids identified and the total signal strength (a proxy for mass) to rank extraction methods. This was followed by a calculation of coefficient of variance to assess the precision of the method [25]. This three-layered structure represented an advance in objective comparison of extraction protocols, which was previously wanting [22]. This represents the strongest way yet found for comparing lipid extraction protocols objectively.

Lastly, the approach to assessing the efficiency of lipid isolation described in this paper is useful because it facilitates choice of extraction method for the sample type at hand. This is increasingly useful, as there is increasing demand for lipidomics, an on a broadening range of sample types. The present assessment characterises the available methods in greater depth. This increases our understanding of the lipidomics tools at our disposal. For example, as this study has shown that fresh milk is a suitable sample type for BuMe [9,10] (developed for plasma) and XMI [25] (developed for dried milk spots) extractions, it shows that fresh milk and either plasma or dried milk spots may be extracted in the same plate easily. They may even be compatible with greater automation. Further research, comprising development of other methods for high-throughput lipidomics, may be useful for expanding our understanding of this process.

## 4. Conclusions

This study was based on the hypothesis that a solvent-based extraction method that comprises a water-wash and the facility to dissolve lipid classes with a range of polarities works best for high-throughput lipidomics. It was found that a lipid extraction method based on dichloromethane and methanol, doped with a lipophilic carbocation (triethylammonium), was the best all-round solvent system. However, this was found not to be mutually exclusive with the performance of other methods in particular areas. There appear to be several methods compatible with milk for high-throughput lipidomics. These observations allow greater insight into the tools available for high-throughput lipidomics on an increasingly broad range of lipid-containing tissue samples.

## 5. Materials and Methods

### 5.1. Ethics

All procedures were conducted in accordance with the UK Home Office Animal (Scientific Procedures) Act 1986 and local ethics committees at Aston University. Animals were maintained at Aston University’s biomedical research facility as described previously [27].

### 5.2. Reagents and Standards

Solvents were purchased from Sigma-Aldrich Ltd. (Gillingham, Dorset, UK) of at least HPLC grade and were not purified further. Lipid standards were purchased from Avanti Polar lipids (Alabaster, AL; through Instruchemie, Delfzijl, NL) and used without purification. Consumables and anonymised pooled human serum were purchased from Sarstedt AG and Co (Leicester, UK) and Thermo Fisher (Hemel Hempstead, Herfordshire, UK). Milk samples were purchased from British supermarkets in 2019.

### 5.3. Sample Processing

The data for this study were acquired in one analytical run of 813 samples, including blank and QC samples. The four examples of heart, liver, milk and serum matrices for measuring coefficient of variance (CV) were prepared as follows. Twelve existing liver and heart homogenates each, from two feeding groups (wither low-protein/high carbohydrate or control), were mixed to make four pooled mixtures each of liver and heart homogenates, prepared as previously described [14,28]. Commercially available, pooled serum was used. Pasteurised Jersey, whole bovine and whole caprine animal milk and soya (*Glycine max.*) drink, purchased from British supermarkets in 2019/2020 and stored at −80 °C, were used. The samples used for comparing lipid extraction methods were prepared as follows. All liver and heart homogenates used above were mixed from all stocks used above. Whole caprine milk and one commercially available human serum were used.

### 5.4. Quality Control

QC samples were used to establish which variables’ signal strength correlated with their concentration. Three QC levels were used, representing 0.25, 0.5 and 1.0× of the total (20 µL). Several reference materials were used, namely, (a) mouse placenta homogenate, (b) mouse liver homogenate, (c) mouse heart homogenate, (d) commercially available pooled human blood serum and (e) whole caprine milk.

### 5.5. Isolation of Lipid Fractions

*DMT—*This procedure was similar to a high-throughput technique described recently [14,21]. Heart homogenate (60 µL), liver homogenate (20 µL), milk (20 µL) and serum (20 µL) samples were placed along with blank and QC samples in the wells of a glass-coated 2.4 mL/well 96w plate (Plate+™, Esslab, Hadleigh, UK). Methanol (150 μL, HPLC grade, spiked with Internal Standards, See Table S1) was added to each of the wells, followed by water (500 µL) and a mixture of solvents (500 µL) comprising dichloromethane and methanol (3:1) doped with triethylammonium chloride (500 mg/L). The mixture was agitated (96 channel pipette) before being centrifuged (3200× *g*, 2 min). A portion of the organic solution (20 µL) was transferred to a high-throughput plate (384w, glass-coated, Esslab Plate+™) before being dried (N_2 (g)_). The dried films were redissolved (TBME, 30 µL/well) and diluted with a stock mixture of alcohols and ammonium acetate (90 µL/well; propan-2-ol:methanol, 2:1; CH_3_COO.NH_4_ 7.5 mM). The analytical plate was heat-sealed and run immediately.

*TBME—*This procedure was as similar as possible to the original protocol for extracting lipids from biological samples [8]. Heart homogenate (60 µL), liver homogenate (20 µL), milk (20 µL) and serum (20 µL) samples were placed along with blank and QC samples in the wells of a glass-coated 2.4 mL/well 96w plate (Plate+™, Esslab, Hadleigh, UK). Methanol (150 μL, HPLC grade, spiked with Internal Standards, See Table S1) was added to each of the wells, followed by water (500 µL) and TBME (500 µL). The mixture was centrifuged (3,200× *g*, 2 min). A portion of the organic solution (20 µL) was transferred to a high-throughput plate (384w, glass-coated, Esslab Plate+™) before being dried (N_2 (g)_). The dried films were redissolved (TBME, 30 µL/well) and diluted with a stock mixture of alcohols and ammonium acetate (90 µL/well; propan-2-ol:methanol, 2:1; CH_3_COO.NH_4_ 7.5 mM). The analytical plate was heat-sealed and run immediately.

*BuMe—*This procedure was as similar as possible to the original protocol for extracting lipids from biological samples [9,10]. Heart homogenate (60 µL), liver homogenate (20 µL), milk (20 µL) and serum (20 µL) samples were placed along with blank and QC samples in the wells of a glass-coated 2.4 mL/well 96w plate (Plate+™, Esslab, Hadleigh, UK). A prepared mixture of methanol (spiked with Internal Standards, See Table S1) and *n*-butanol (1:1, 200 µL) was added to each of the wells and agitated until homogenous. A portion of the mixture (20 µL) was transferred to a shallow 96w plate before being dried (N_2 (g)_). The dried films were redissolved (TBME, 30 µL/well) and diluted with a stock mixture of alcohols and ammonium acetate (90 µL/well; propan-2-ol:methanol, 2:1; CH_3_COO.NH_4_ 7.5 mM) before being transferred to a high-throughput plate (384w, glass-coated, Esslab Plate+™) before being dried (N_2 (g)_). The analytical plate was heat-sealed and run immediately.

*XMI*—This procedure has not been described before. Heart homogenate (60 µL), liver homogenate (20 µL), milk (20 µL) and serum (20 µL) samples were placed along with blank and QC samples in the wells of a glass-coated 2.4 mL/well 96w plate (Plate+™, Esslab, Hadleigh, UK). A prepared mixture of solvents and xylene/methanol/isopropanol (1:2:4, 500 µL, methanol spiked with Internal Standards, See Table S1) was added to each of the wells and agitated until homogenous. A portion of the mixture (20 µL) was transferred to a shallow 96w plate before being dried (N_2 (g)_). The dried films were redissolved (TBME, 30 µL/well) and diluted with a stock mixture of alcohols and ammonium acetate (90 µL/well; propan-2-ol:methanol, 2:1; CH_3_COO.NH_4_ 7.5 mM) before being transferred to a high-throughput plate (384w, glass-coated, Esslab Plate+™) before being dried (N_2 (g)_). The analytical plate was heat-sealed and run immediately.

### 5.6. Mass Spectrometry

*Instrument—*Samples were infused into an Exactive Orbitrap (Thermo, Hemel Hampstead, UK), using a Triversa Nanomate (Advion, Ithaca US). Samples were ionised at 1.2 kV in the positive ion mode. The Exactive started acquiring data 20 s after sample aspiration began. After 72 s of acquisition in positive mode, the Nanomate and the Exactive switched over to negative mode, decreasing the voltage to −1.5 kV. The spray was maintained for another 66 s, after which the analysis was stopped and the tip discarded, before the analysis of the next sample. The sample plate was kept at 15 °C throughout the acquisition. Samples were run in row order.

*Data processing—*Raw high-resolution mass-spectrometry data were processed using XCMS (www.bioconductor.org) and Peakpicker v 2.0 (an in-house R script [24]). Lists of known species (by *m/z*) were used for both positive ion and negative ion mode (~8.5k species). Variables whose mass deviated by more than 9 ppm from the expected value had a signal/noise ratio of <3 and had signals for fewer than 50% of all samples that were discarded. The correlation of signal intensity to concentration of human placenta, mouse liver, human serum and pooled human seminal plasma samples as QCs (0.25, 0.5, 1.0×) was used to identify the lipid signals, the strength of which was linearly proportional to abundance (threshold for acceptance was a correlation of 0.75). Remaining signals (passes) were then divided by the sum of signals for that sample and expressed per mille (‰). Each *m/z* signal identified was interpreted as a given isoform of a lipid with an appropriate adduct for that *m/z*. Like isoforms with different adducts were not summed. Zero values were interpreted as not measured. All statistical calculations were done on these finalised values.

### 5.7. Statistical Analyses

The analysis was structured according to a prepared analysis plan. Uni- and bivariate analyses were carried out using Excel 2016. Multivariate analyses were run using MetaboAnalyst 4.0 [29]. Abundance of lipid(s) is shown as mean ± standard deviation unless otherwise stated.

## Figures and Tables

**Table 1:** Variables stratified by parameters for chemical structures. The proportion (%) of variables in strata of variance for each of four matrices. Coefficient of variance calculated from the standard deviation of 16-20 samples of each of four groups, divided by the mean for the same group. Milk samples and human serum were drawn from four different commercial sources (Milk1, un-homogenised Jersey milk; Milk2, whole caprine milk; Milk3, soya (*Glycine max*) drink; Milk4, whole, homogenised bovine milk). Murine heart and liver samples were drawn from two pools of two feeding phenotypes.

	Fraction of Variables (%)							
	+ve Ionisation Mode				−ve Ionisation Mode			
CV	Serum	Milk	Liver	Heart	Serum	Milk	Liver	Heart
0–10%	19	37	20	28	19	6	26	40
10–20%	16	10	20	20	17	7	16	18
20–30%	11	3	7	6	13	5	13	11
30–50%	14	5	10	10	18	13	16	13
>50%	41	45	43	36	33	69	28	19
**Total**	**100%, 197 variables**				**100%, 273 variables**			

## Supplementary Materials

Mass Spectrometry signals sheets and Table S1: List of internal lipid standards, are available at https://www.mdpi.com/1420-3049/25/14/3192/s1.
