# Supplementary material for: Extraction of Lipids from Liquid Biological Samples for High-Throughput Lipidomics
Source: Molecules. 2020 Jul 13;25(14):3192. doi: 10.3390/molecules25143192 (PMC7397209; doi:10.3390/molecules25143192)
Supplement: Supplementary file 1 [file molecules-25-03192-s001.zip › Furse et al 2020_Extraction of lipids for high throughput SI_2020-06-08.docx]

Supplementary Tables and Figures for ‘Extraction of lipids from liquid biological samples for high throughput lipidomics’, Furse *et al.*

Supplementary Tables

| **Lipid Class** | **Isoform** | **Mass** | **Stock concentration (μM)** |
| --- | --- | --- | --- |
| Cholesteryl ester | CE(18:0*-d*_6_ ) | 658·6603 | 3·80 |
| Ceramide | C16*-d*_31_ Ceramide | 566·6928 | 4·40 |
| Fatty acid | C15:0*-d*_29_ FA | 271·4076 | 9·23 |
| Fatty acid | C17:0*-d*_33_ FA | 303·4635 | 8·25 |
| Fatty acid | C20:0*-d*_39_ FA | 351·5484 | 7·13 |
| *lyso-*Phosphatidylcholine | *lyso-*PC(C14:0)*-d*_42_ | 421·5481 | 5·93 |
| Phosphatidic acid | PA(C16:0*-d*_31_/C18:1) Na^+^ salt | 704·6784 | 3·55 |
| Phosphatidylcholine | PC(C16:0*-d*_31_/C18:1) | 789·7637 | 3·18 |
| Phosphatidylethanolamine | PE(C16:0*-d*_31_/C18:1) | 747·7171 | 3·35 |
| Phosphatidylglycerol | PG(C16:0*-d*_31_/C18:1) Na^+^ salt | 778·7157 | 3·20 |
| Phosphatidylinositol | PI(C16:0*-d*_31_/C18:1) NH_4_^+^ salt | 881·7441 | 1·13 |
| Phosphatidylserine | PS(C16:0*-d*_62_) Na^+^ salt | 733·7794 | 3·40 |
| Sphingomyelin | SM(C16:0*-d*_31_) | 733·7603 | 2·04 |
| Triglyceride | TG(45:0*-d*_29_) | 852·2314 | 2·93 |
| Triglyceride | TG(48:0*-d*_31_) | 900·3167 | 2·78 |
| Triglyceride | TG(54:0*-d*_35_) | 996·4851 | 2·50 |

Supplementary Table 1. List of internal standards used for lipid profiling in the present study
